# VEGF_165_b, an antiangiogenic VEGF-A isoform, binds and inhibits bevacizumab treatment in experimental colorectal carcinoma: balance of pro- and antiangiogenic VEGF-A isoforms has implications for therapy

**DOI:** 10.1038/sj.bjc.6604308

**Published:** 2008-03-18

**Authors:** A H R Varey, E S Rennel, Y Qiu, H S Bevan, R M Perrin, S Raffy, A R Dixon, C Paraskeva, O Zaccheo, A B Hassan, S J Harper, D O Bates

**Affiliations:** 1 Microvascular Research Laboratories, Department of Physiology and Pharmacology, School of Veterinary Sciences, University of Bristol, Bristol, UK; 2 Department of Surgery, Frenchay Hospital, Bristol, UK; 3 Department of Cellular and Molecular Medicine, University of Bristol, Bristol, UK

**Keywords:** bevacizumab, VEGF, VEGF_165_b, biomarker, angiogenesis, colon carcinoma

## Abstract

Bevacizumab, an anti-vascular endothelial growth factor (VEGF-A) antibody, is used in metastatic colorectal carcinoma (CRC) treatment, but responses are unpredictable. Vascular endothelial growth factor is alternatively spliced to form proangiogenic VEGF_165_ and antiangiogenic VEGF_165_b. Using isoform-specific enzyme-linked immunosorbent assay and quantitative polymerase chain reaction, we found that over 90% of the VEGF in normal colonic tissue was VEGF_xxx_b, but there was a variable upregulation of VEGF_xxx_ and downregulation of VEGF_xxx_b in paired human CRC samples. Furthermore, cultured colonic adenoma cells expressed predominantly VEGF_xxx_b, whereas colonic carcinoma cells expressed predominantly VEGF_xxx_. However, adenoma cells exposed to hypoxia switched their expression from predominantly VEGF_xxx_b to predominantly VEGF_xxx_. VEGF_165_b overexpression in LS174t colon cancer cells inhibited colon carcinoma growth in mouse xenograft models. Western blotting and surface plasmon resonance showed that VEGF_165_b bound to bevacizumab with similar affinity as VEGF_165_. However, although bevacizumab effectively inhibited the rapid growth of colon carcinomas expressing VEGF_165_, it did not affect the slower growth of tumours from colonic carcinoma cells expressing VEGF_165_b. Both bevacizumab and anti-VEGF_165_b-specific antibodies were cytotoxic to colonic epithelial cells, but less so to colonic carcinoma cells. These results show that the balance of antiangiogenic to proangiogenic isoforms switches to a variable extent in CRC, regulates tumour growth rates and affects the sensitivity of tumours to bevacizumab by competitive binding. Together with the identification of an autocrine cytoprotective role for VEGF_165_b in colonic epithelial cells, these results indicate that bevacizumab treatment of human CRC may depend upon this balance of VEGF isoforms.

Solid tumour growth is dependent on the induction of their own blood supply by inducing a proangiogenic state in the tissue environment, regulating this balance between proangiogenic growth factors and antiangiogenic inhibitors (Folkman, 1985, 1995; Boehm *et al*, 1997). One growth factor that has been shown to be an effective target for antiangiogenic therapy (AAT) is vascular endothelial growth factor-A (VEGF-A). Inhibition of VEGF by humanised monoclonal antibodies has been shown to be effective in increasing the median survival in metastatic colorectal cancer (CRC) when combined with chemotherapy (Hurwitz *et al*, 2004).

Vascular endothelial growth factor-A is generated by alternative splicing from eight exons within the *VEGF-A* gene. All isoforms contain exons 1–5 and the terminal exon, exon 8. Exons 6 and 7, which encode heparin-binding domains, can be included or excluded. This gives rise to a family of proteins termed according to their amino-acid number, VEGF_165_, VEGF_121_, VEGF_189_ and so on. Exon 8, however, contains two 3′ splice sites in the nucleotide sequences, which can be used by the cell to generate two families of isoforms with identical length, but differing C-terminal amino-acid sequences (Bates *et al*, 2002). VEGF_xxx_, the proangiogenic family of isoforms, is generated by use of the most proximal sequence in exon 8 (resulting from inclusion of exon 8a). The more recently described VEGF_xxx_b isoforms are generated by the use of a distal splice site, 66 bp further along the gene from the proximal splice site. This results in splicing out of exon 8a and the production of mRNA sequences that encode the VEGF_xxx_b family (Bates *et al*, 2002). The two resultant families of proteins are of the same length, but with different carboxyl termini. VEGF_165_b was the first of these exon 8b-encoded isoforms identified and subsequent studies demonstrated the existence of VEGF_121_b, VEGF_183_b, VEGF_145_b (Perrin *et al*, 2005) and VEGF_189_b (Miller-Kasprzak and Jagodzinski, 2008).

The functional consequences of this altered C terminus are that VEGF_165_b homodimers compete with VEGF_165_ homodimers for binding to their principal receptor, VEGFR-2, at a one-to-one ratio and inhibit endothelial cell proliferation and migration in culture (Woolard *et al*, 2004; Cebe Suarez *et al*, 2006). VEGF_165_b blocks VEGF_165_-driven angiogenesis *in vivo* in the rabbit, rat (Woolard *et al*, 2004), mouse and chick (Cebe Suarez *et al*, 2006), and human malignant melanomas consisting of cells overexpressing VEGF_165_b and cells expressing VEGF_165_ grow slower in nude mice than those consisting of cells expressing VEGF_165_ alone. Recombinant human VEGF_165_b is also antiangiogenic in hypoxia-driven angiogenesis in the eye (Konopatskaya *et al*, 2006).

Although VEGF has been shown to be critical in CRC by inhibition studies, the expression of VEGF in CRC has not been investigated using tools that distinguish between the proangiogenic VEGF_xxx_ isoforms and the antiangiogenic VEGF_xxx_b isoforms. The vast majority of studies have measured total VEGF levels in plasma, tumours or serum using commercially available antibodies that do not distinguish between pro- and antiangiogenic isoforms, as commercial enzyme-linked immunosorbent assays (ELISAs) detect VEGF_xxx_b isoforms. Furthermore, it is not known whether the VEGF_xxx_b isoforms are able to slow or reduce tumour growth if they are highly expressed. To determine whether this antiangiogenic isoform family is expressed in CRC, and how that expression may be regulated, this study compares the balance of expression of the VEGF_xxx_b family of isoforms in human CRC with paired normal colonic mucosa samples and show how the expression of VEGF_xxx_b and VEGF_xxx_ is altered during the malignant transformation of colonic adenoma cells *in vitro*. Furthermore, to determine whether this balance has the potential to regulate tumour growth rates, we have measured VEGF_165_b functional effects on colon carcinoma growth in animal models where the tumour growth was VEGF-dependent. Furthermore, we assess whether bevacizumab either specifically binds VEGF_xxx_ (proangiogenic) isoforms, or also has cross-reactivity with antiangiogenic VEGF_165_b and whether it inhibits VEGF_165_b-expressing tumours.

## MATERIALS AND METHODS

### Human tissue samples

Paired colon samples were from partial colon resection for carcinoma. Samples were obtained by taking biopsies of the fresh specimen from a nonnecrotic central portion of the tumour and from a peripheral part of the macroscopically normal colonic epithelium (*n*=18 pairs). Samples were collected with Local Ethics committee approval. The mean patient age was 71.5 (range 58–80) years, with 62% male subjects and Duke's staging as follows: 6.7% A, 46.7% B and 46.7% C. Biopsies were immediately frozen in liquid nitrogen and then stored at −80°C until processed. Biopsies were frozen in liquid nitrogen again immediately prior to manual slicing with a sterile blade. The mass of each tissue was recorded, and samples were homogenised and mRNA and protein extracted as described below.

### Expression analysis

For each of eight pairs of samples, mRNA was extracted from approximately 200 mg of tissue (Chomczynski and Sacchi, 1987) and reverse transcribed as previously described (Bates *et al*, 2002). The cDNA was amplified using primers complementary to VEGF exon 7 and the 3′-UTR downstream of exon 8b, as previously described (Bates *et al*, 2002). The products were subjected to standard agarose gel electrophoresis and ethidium bromide staining. Protein was extracted from approximately 250 mg tissue, from 18 pairs of samples, resolved by 12% sodium dodecyl sulphate-polyacrylamide gel electrophoresis, transferred and immunoblotted as previously described (Woolard *et al*, 2004). Briefly, membranes containing recombinant human VEGF_165_ and/or VEGF_165_b protein (50 ng of each) and protein samples extracted from colon (100 *μ*g of each) were probed with mouse anti-VEGF_xxx_b IgG (2 *μ*g ml^−1^ A56/1; R&D Systems, cat no. MAB3045) (Woolard *et al*, 2004) or rabbit anti-VEGF IgG (1 *μ*g ml^−1^ A-20; Santa Cruz Biotechnology, Santa Cruz, CA, USA) and detected with horseradish peroxidase-conjugated stabilised goat anti-mouse or anti-rabbit IgG (1/7000; Pierce Biotechnology, Rockford, IL, USA). Visualisation of protein bands was achieved with SuperSignal West Femto Maximum Sensitivity Substrate Kit (Pierce Biotechnology).

### Quantitative RT-PCR

Quantitative PCR assays were carried out on cDNA generated as above. An exon 7b forward primer and a 3′-UTR primer (both pan-VEGF quantitative polymerase chain reaction (Q-PCR) from Primer Design, Southampton, UK) or an exon 7a forward primer 5′-TTGCTCAGAGCGGAGAAAGC-3′and a reverse primer specific for exon 8a that did not detect VEGF_xxx_b isoforms 5′-TCACCGCCTCGGCTTGTCACAT-3′ were used. Reactions were performed using a SmartCyclerII (Cepheid, Sunnyvale, CA, USA) q-PCR machine, with 25 *μ*l reaction volumes comprising 12.5 *μ*l Quantitect SYBRgreen 2 × master mix (Qiagen, Crawley, UK), 1 *μ*l cDNA and 1 *μ*l primer mix cycled at 95°C for 15 min followed by 50 cycles of 60°C for 30 s, 72°C for 60 s, 79°C for 15 s (reading) and 95°C for 30 s. A melt curve was then performed by ramping the temperature from 60 to 95°C at 0.2°C per second, reading throughout. DNA standards used were VEGF_165_b or VEGF_165_ cloned into pcDNA3, or oligonucleotides containing the full sequence between the primers (Primer Design).

**Figure 1:**
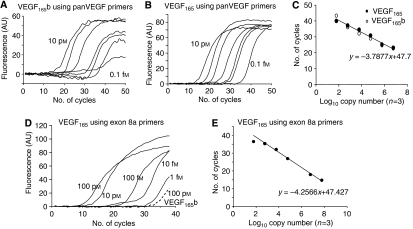
Quantification of mRNA expression of pan-VEGF and VEGF_xxx_ isoform mRNA by Q-PCR. (**A** and **B**) Primers that detected all isoforms were used to detect increasing amounts of VEGF_165_b (**A**) or VEGF_165_ (**B**). (**C**) Standard curves for the two isoforms were the same indicating that a mixture of both could be assessed equally. (**D**) Amplification of VEGF_xxx_ cDNA specifically. Primers specific to exon 8a resulted in amplification that was more than six orders of magnitude more sensitive for VEGF_165_ than VEGF_165_b (dotted line). (**E**) Standard curve using VEGF_165_ as a template.

Figures 1A and B show examples of the reverse transcription-polymerase chain reaction (RT-PCR) curves for VEGF_165_b and VEGF_165_ templates respectively using pan-VEGF primers. Figure 1C shows the standard curve generated from cycle threshold for the two templates, showing that there was no difference in the standard curves (*n*=3). Thus the efficiency of amplification of the two templates is not different. Figure 1D shows the amplification curves for the exon 8a primers using the VEGF_165_ sequence as a template (VEGF_165_b template did not result in amplification until 18 cycles later than equivalent VEGF_165_ concentration). Total VEGF and VEGF_xxx_ copy numbers were calculated for each sample using the calibration curve shown in Figure 1E. The difference between the total VEGF and the VEGF_xxx_ copy number was assumed to be the VEGF_165_b copy number.

### Immunohistochemistry

Immunohistochemistry was performed on formalin-fixed paraffin-embedded normal colonic mucosa, obtained with local ethics committee approval from archival material as previously described (Woolard *et al*, 2004). Sections were stained with either mouse monoclonal anti-VEGF_xxx_b IgG (8 *μ*g ml^−1^; R&D Systems, cat no. MAB3045) or 5 *μ*g ml^−1^ mouse anti-VEGF IgG (Santa Cruz; SC-7269) or a normal mouse IgG (Sigma Aldrich, Gillingham, UK; I8765), as a negative control.

### ELISAs

pan-VEGF mouse capture antibody (0.1 *μ*g) (Duoset VEGF ELISA DY-293; R&D Systems, Minneapolis, MN, USA) diluted in 1 × phosphate-buffered saline (PBS) (pH 7.4) was adsorbed onto each well of a 96-well sterile plate (Immulon 2HB Thermo Life Sciences, Basingstoke, UK) or, for the VEGF_xxx_b ELISA, 0.08 *μ*g AF293-NA Goat anti-VEGF polyclonal IgG (R&D Systems), overnight at room temperature. The plates were washed three times between each step with 1 × PBS-Tween (0.05%). After blocking with bovine serum albumin in PBS for 30 min at 37°C, recombinant human VEGF_165_ standards or VEGF_165_b (R&D Systems) diluted in blocking solution (ranging from 62.5 pg ml^−1^ to 2 ng ml^−1^) or protein sample was added to each well. After incubation for 30 min at 37°C with shaking and three washes, 100 *μ*l of biotinylated goat anti-human VEGF (50 ng ml^−1^ in blocking solution; R&D Systems) or a mouse monoclonal anti-VEGF_xxx_b-biotinylated IgG (clone 264610/1; R&D Systems) at 0.4 *μ*g ml^−1^ was added to each well, and plates left for 30 min at 37°C with shaking. Streptavidin-HRP (100 *μ*l) (R&D Systems) at 1 : 200 dilution in PBS was added, plates left at room temperature for 20 min and 100 *μ*l per well *O-*phenylenediamine dihydrochloride solution (Substrate reagent pack DY-999; R&D Systems) added, protected from light and incubated for 20 min at room temperature. The reaction was stopped with 100 *μ*l per well 1 mol l^−1^ H_2_SO_4_ (10276; BDH Chemicals, Poole, UK), and absorbance read immediately in an Opsys MR 96-well plate reader (Dynex Technologies, Chantilly, VA, USA) at 492 nm, with control reading at 460 nm.

### Characterisation of the ELISA

**Figure 2:**
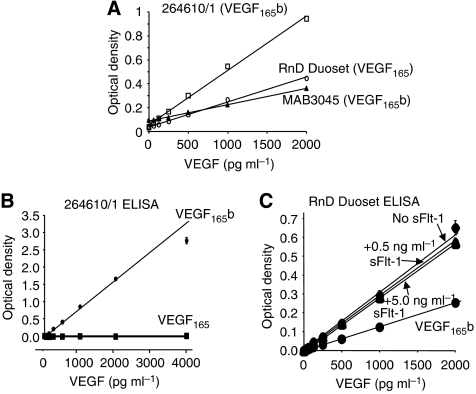
Quantification of VEGF_xxx_b protein expression by ELISA. (**A**) Sensitivity of VEGF_165_b-specific antibody 264610/1 was determined using a sandwich ELISA. Increasing concentrations of VEGF_165_b (for clone 264610/1 and MAB3045) or VEGF_165_ (for R&D DuoSet, R&D Systems, Minneapolis, MN, USA) were incubated with a goat anti-VEGF capture antibody, then detected by either biotinylated 264610/1 mouse anti-VEGF (R&D Duoset) or MAB3045 and a biotinylated secondary antibody. (**B**) Specificity of the VEGF_165_b ELISA using biotinylated 264610/1 was determined by incubating increasing concentrations of human recombinant VEGF_165_b, or VEGF_165_ with a pan-VEGF capture antibody adhered to the plate, then detecting with biotinylated 264610/1. (**C**) The commercially available ELISA is less sensitive for VEGF_165_b than for VEGF_165_. Increasing concentrations of protein (determined by Bradford Assay) were incubated with a mouse monoclonal capture antibody and detected with a biotinylated mouse-pan-VEGF capture antibody. VEGF_165_b was underestimated by 42%. The lower affinity of the capture antibody for VEGF_165_b was confirmed by Biacore. sFlt-1 did not interfere with this ELISA.

We used both this antibody (MAB3045) and a separate antibody generated against the terminal nine amino acids of VEGF_165_b, raised by R&D Systems and biotinylated (clone 264610/1), to quantitate the relative levels of VEGF_165_b. Both VEGF_165_b antibodies were as sensitive as the commercially available VEGF ELISA (see Figure 2A). The biotinylated 264610/1 antibody specifically detects VEGF_165_b, is accurate down to below 62.5 pg ml^−1^ VEGF_165_b and does not detect VEGF_165_, even at 4 ng ml^−1^ in an ELISA (Figure 2B). Both antibodies were used to determine the amount of VEGF_xxx_b in human tissues from pancreatic islets, placenta, lung, colon and prostate. The two ELISAs did not differ in their results (e.g., colon tissue, 130±40 pg mg^−1^ MAB3045 ELISA 128±20 pg mg^−1^ clone 264610/1 ELISA, *N*=18). The biotinylated 264610 ELISA was used to quantitate the amount of VEGF in tissue samples, as it was more sensitive, a simpler procedure, could use commercially available capture antibodies and the protocol was most comparable to the commercial VEGF DuoSet ELISA. To determine whether VEGF_165_ could interfere with this VEGF_165_b ELISA, serial dilutions of rhVEGF**_165_** were assayed. As seen in Figure 2B, there was no significant change in OD values by the addition of rhVEGF**_165_** as high as 4000 pg ml^−1^, indicating that the VEGF_xxx_b ELISA specifically detects VEGF_165_b, and is not affected by the conventional VEGF isoform, VEGF_165_.

To determine whether commercially available ELISAs also detected VEGF_165_b, we carried out a VEGF ELISA using increasing concentrations of VEGF_165_b. Figure 2C shows that increasing concentrations of VEGF_165_b were detected by the R&D Duoset kit – the most widely used ELISA. Interestingly, this ELISA detects VEGF_165_b at a lower affinity than VEGF_165_. The ratio of the slopes is 0.42±0.004 or 42±0.4%. To confirm this, we used VEGF_165_b generated from two different sources – R&D Systems and an in-house production (both proteins were quantitated by Bradford Assay). The R&D Duoset Kit (DY293B) is a second generation ELISA introduced in 2004. We are unaware of published information on sFlt-1 interference in the current DuoSet kit. Figure 2C shows that increasing concentrations of sFlt-1 did not affect the pan-VEGF ELISA at least up to 2000 pg ml^−1^ VEGF.

With the previous ELISA, VEGF_165_b levels were detected at 100% of VEGF_165_, indicating that the previous ELISA kit had the same affinity for both isoforms. To ensure that this was due to a difference in affinity of the antibodies for the two isoforms, we carried out surface plasmon resonance analysis of binding coefficients.

### Surface plasmon resonance

**Figure 3:**
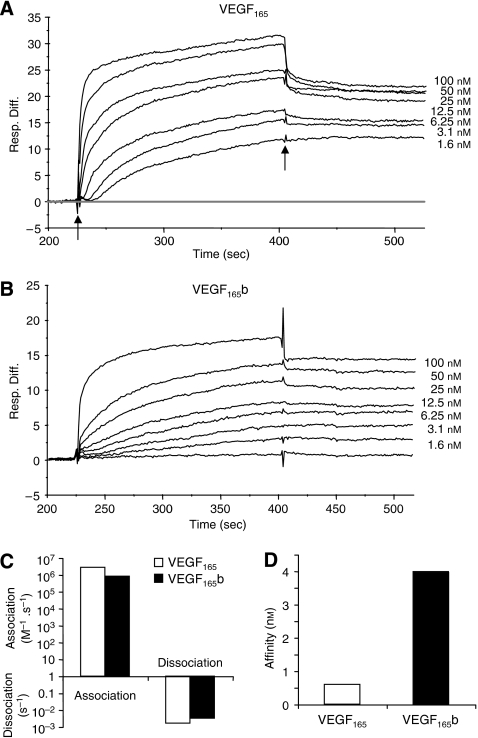
The commercially available VEGF antibody has a lower affinity for VEGF_165_b than VEGF_165_. The mouse detection antibody from the R&D ELISA Duoset was immobilised on a sensor chip, and VEGF isoforms allowed to flow over the detecting chip. (**A**) VEGF_165_ resulted in an increase in signal upon loading (arrow 1), which reduced on replacement with wash solution (arrow 2). (**B**) With the same set up, VEGF_165_b was also detected, but the response rate was slower, and the release was faster. (**C**) Association and disassociation coefficients calculated using Langmuir 1 : 1 binding analysis. (**D**) Affinity coefficient calculated from the dissociation and association constants measured. The antibody has approximately an 6.6-fold weaker affinity for VEGF_165_b than VEGF_165_.

To compare the binding affinities of VEGF_165_ and VEGF_165_b to the pan-VEGF antibody used in the R&D Duoset detection kit, we amine-coupled the latter to a CM5 sensor chip (Biacore AB, Uppsala, Sweden) to an immobilisation level of 630 response units (RU). To compare the binding affinities of VEGF_165_ and VEGF_165_b to bevacizumab, the latter was amine-coupled to a CM5 sensor chip (Biacore AB) to an immobilisation level of 580 RU. The coupling was performed using EDC/NHS and 1 mol l^−1^ ethanolamine (Biacore) as per the manufacturer's instructions, with the bevacizumab dissolved in 10 mmol l^−1^ sodium acetate (pH 4.5). A blank reference cell was formed by the same activation and deactivation process involved in amine coupling without adding antibody. Samples containing VEGF_165_ or VEGF_165_b diluted in HBS-EP sample buffer (Hepes-buffered saline with EDTA and P20 surfactant, Biacore AB) were then run at twofold serial dilutions from 180 nmol l^−1^ down, in random order in duplicate. Injection was performed at 30 *μ*l min^−1^ for 3 min, followed by 6 min of buffer only, for monitoring of dissociation. Regeneration between each interaction was performed by injection of 4 mol l^−1^ MgCl_2_ at 20 *μ*l min^−1^ for 40 s, followed by a 2-min period of stabilisation before the next injection. Figure 3A shows the binding curves of VEGF_165_ to the RnD detection antibody and Figure 3B shows binding of VEGF_165_b to the same antibody. The RnD detection antibody had a higher association coefficient for VEGF_165_ than VEGF_165_b and a lower dissociation coefficient for VEGF_165_ than VEGF_165_b (Figure 3C), resulting in an affinity of 602 pM for VEGF_165_, but 3.98 nM for VEGF_165_b, an ∼6.6-fold difference in affinity, indicating that the underestimation of the commercial ELISA for VEGF_165_b was due to a difference in affinity for the antigen.

The actual VEGF concentrations (VEGF_total_) in human tissue are the sum of VEGF_xxx_ and VEGF_xxx_b. 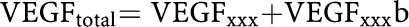 The commercially available pan-VEGF ELISA has a lower affinity for VEGF_165_b than for VEGF_165_ by 42%. Therefore the measured VEGF levels (VEGF_measured_) in the commercially available ELISA are the sum of VEGF_165_ and 42% of VEGF_165_b. 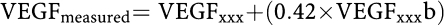


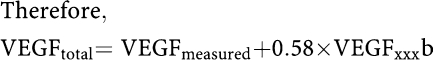
 We used the above correction to estimate tissue concentration of VEGF_xxx_b and its relative proportion of total VEGF.

### Adenoma-carcinoma cell VEGF expression

Colonic adenoma cells (AAC1) and their *in vitro*-derived carcinoma cells (10C) were kindly donated by Professor C Paraskeva (Williams *et al*, 1990). The adenoma cells are a non-tumour-forming clonogenic variant of the PC/AA cell line derived from a polyp from a patient with familial adenoma polyposis (Paraskeva *et al*, 1988). The cells were cultured to 100% confluence in Dulbecco's modified Eagle's medium supplemented with 20% fetal bovine serum, 1% penicillin/streptomycin and 0.2% Actrapid insulin (Novo Nordisk, Crawley, UK). Protein was extracted from confluent cells in fresh media as above cells were placed in a hypoxic chamber (Billups-Rothenburg, Del Mar, CA, USA) for 5 min at 20 l min^−1^ with 5% CO_2_/nitrogen gas mixture (BOC) and incubated at 37°C. The gas was changed twice per day.

### *In vivo* tumour model

LS174t human colon carcinoma cell lines were used (ECACC, Salisbury, UK) (Yuan *et al*, 1996; Lee *et al*, 2000). Cells were transfected with 1 *μ*g of purified plasmid pcDNA3 either as empty vector, with VEGF_165_, VEGF_165_b, or both VEGF_165_ and VEGF_165_b vectors, using Lipofectamine Plus (Invitrogen, Carlsbad, CA, USA) as per the manufacturer's instructions. Cells were selected using geneticin (500 *μ*g ml^−1^). Conditioned media were analysed by ELISAs for VEGF and VEGF_xxx_b production per cell per 24 h, to confirm expression levels of VEGF isoforms. A total of 2 × 10^6^ cells were injected subcutaneously into the lumbar region of nude mice (six per group unless otherwise stated). Mice were then monitored every 2–3 days and tumour length and width measured. When the first tumour reached 16 mm in maximum diameter, all mice were killed. Tumour volumes were calculated according to the formula (length × width × (length+width)/2). Injections, measurements and analysis were all carried out with the investigators blinded to group.

### Cytotoxicity

Measurement of the cell-cytotoxicity effects of blocking VEGF isoforms was performed using a lactate dehydrogenase (LDH) assay following exposure of the cells to various antibodies and RTKIs as described in the results. Cells were grown to 90–100% confluence in 96-well plates prior to treatment in serum-free medium, against which treatment was compared. A Cytotox 96 Non-Radioactive Cytotoxicity Assay (Promega Corp, Madison, WI, USA, no. G1780) was used according to the manufacturer's instructions.

### Statistics

Means and standard errors are given unless stated otherwise. Tumour volumes, cell cycle parameters and apoptosis were compared by one-way analysis of variance (ANOVA) followed by a Bonferroni *post-hoc* test. Tumour growth curves were fitted by nonlinear regression using an exponential curve fit in Prism. Doubling times were calculated from 0.69 k^−1^, and are given as mean (95% confidence intervals (CI)), and curve-fitting parameters compared using an F-test. Analysis of ELISA results was performed using Wilcoxon's signed matched ranks at 95% significance level (two-tailed).

## RESULTS

### Normal colonic epithelial cells and colonic carcinomas expressed VEGF_165_b mRNA

**Figure 4:**
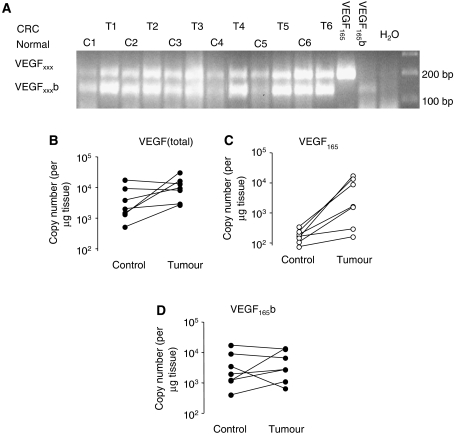
VEGF_165_b mRNA is expressed in human colon tissue and colon cancer. (**A**) VEGF_xxx_b mRNA is expressed in normal and cancerous colon. PCR of cDNA reverse transcribed from RNA extracted from paired human colon samples shows two bands, the proximal splice isoforms (VEGF_xxx_, ∼200 bp) and the distal splice isoforms (VEGF_xxx_b, ∼135 bp). (**B**–**D**) Q-PCR for pan-VEGF (VEGF_165_b and VEGF_165_) and VEGF_165_ isoforms. (**B**) Primers that detected all 165 amino acid-coding isoforms were used to detect increasing amounts of total VEGF (VEGF_165_b and VEGF_165_). (**C**) Exon 8a-specific primers were used to measure the amount of VEGF_165_, which was significantly increased in colon cancers, *P*<0.01. (**D**) VEGF_165_b levels calculated from the VEGF_165_ and total VEGF levels.

To determine whether VEGF_165_b and VEGF_165_ mRNA were expressed in normal and cancerous colon, RT-PCR using primers that distinguish between the two families of isoforms was carried out on eight pairs of samples. Reverse transcription-polymerase chain reaction gave two bands, one at ∼135 bp, consistent with VEGF_165_b or VEGF_189_b, and one at ∼200 bp, consistent with VEGF_165_ and VEGF_189_. This size difference was due to the splicing out of exon 8a in the VEGF_xxx_b family, resulting in the shorter mRNA (although exon 8b is present in the mRNA of the VEGF_xxx_ family, a stop codon in exon 8a prevents its translation). VEGF_xxx_ and VEGF_xxx_b mRNA expression was detected in both normal and tumour tissue (Figure 4A).

### VEGF mRNA is differentially spliced in colon cancer

Quantitative PCR on mRNA extracted from seven pairs of colorectal normal and tumour tissue demonstrated that the VEGF_xxx_ mRNA copy number was only 9.1±2.8% of the total VEGF level in normal tissues, indicating that VEGF_xxx_b species form more than 90% of the mRNA. There was an increase in copy number of all VEGF isoforms from 5±2.2 to 11±3.5 × 10^3^ copies per *μ*g of tissue (Figure 4B). This upregulation was specific for the VEGF_165_ isoform (0.18±0.32 to 6.0±2.6 × 10^3^ copies per *μ*g, Figure 4C, *P*<0.01, ANOVA), and not for the VEGF_165_b isoform (4.8±2.2 to 5.6±2.0 × 10^3^ copies per *μ*g, Figure 4D), such that in cancers, 45±13% of the VEGF mRNA was VEGF_165_.

### VEGF_165_b and VEGF_121_b protein are expressed in normal and cancerous colon

**Figure 5:**
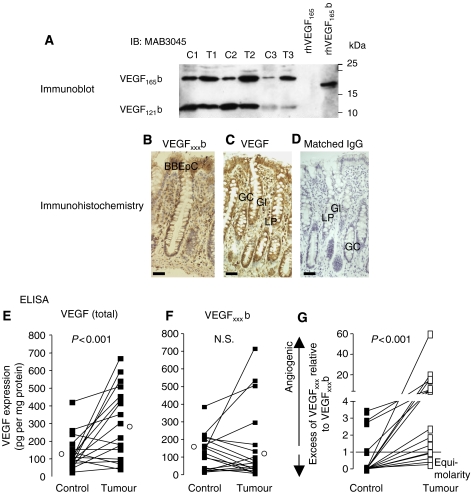
VEGF_xxx_b is the predominant family of VEGF proteins in normal colon but not in colon cancer. (**A**) Western blots of VEGF_xxx_b expression in normal and cancerous colon. Two strong bands are seen in each tissue, consistent with VEGF_165_b and VEGF_121_b. Longer isoforms are very faintly visible, indicating VEGF_183_b and VEGF_189_b. C=control, T=tumour, rhVEGF_165_=recombinant human VEGF_165_, VEGF_165_b=recombinant human VEGF_165_b (unglycosylated). (**B**–**D**) Immunohistochemical staining of normal colonic mucosa. (**B**) Staining with an antibody that recognises only the VEGF_xxx_b isoforms. (**C**) Staining with an antibody that recognises all VEGF isoforms. (**D**) Control IgG stain. Vascular endothelial growth factor and VEGF_xxx_b were localised to the brush border epithelial cells (BBEpC), goblet cells (GC) of the intestinal glands (Gl) and to some, but not all, plasma cells of the lamina propria (LP). Scale bar=50 *μ*m. (**E**–**G**) Measurement of total VEGF (**E**) and VEGF_xxx_b (**F**) expression in normal and cancerous colon. VEGF_xxx_ but not VEGF_xxx_b, was upregulated in colorectal carcinoma mean values shown by open circles. (**G**) VEGF_xxx_ is present in excess over VEGF_xxx_b in CRC, but not in normal tissue. Excess VEGF_xxx_ (values greater than 1) results in a proangiogenic environment, whereas excess VEGF_xxx_b (values less than 1) results in an antiangiogenic environment.

To determine whether protein expression of VEGF_xxx_b isoforms was present in normal and tumour tissues, western blotting and ELISA were carried out using antibodies specific to VEGF_165_b and antibodies that do not distinguish between isoforms. The previously characterised anti-VEGF_165_b antibody (R&D Systems, cat no. MAB3045) demonstrates highly specific binding to recombinant human VEGF_165_b but not to recombinant human VEGF_165_ (Pritchard-Jones *et al*, 2007). Figure 5A shows that both VEGF_121_b and VEGF_165_b were expressed in both normal colonic mucosa and in carcinomas as detected by western blotting. To determine which cells in colonic epithelium expressed the VEGF_xxx_b isoforms, we used immunohistochemistry to stain the normal colon samples. Figure 5B shows that VEGF_xxx_b was highly expressed in colonic epithelial cells of the brush border (especially cells at the apex of the villi), cells within the lamina propria with plasma cell morphology and goblet cells. Staining of sections with the VEGF antibody that recognises all VEGF isoforms (including VEGF_xxx_b isoforms, Figure 5C) showed that VEGF was expressed in all the places where VEGF_165_b staining was apparent. Nonspecific isotype control IgG did not stain (Figure 5D).

### Increased VEGF protein levels in cancer are restricted to the angiogenic isoforms

To determine whether the previously reported increase in VEGF expression (Ellis *et al*, 2000; Reinmuth *et al*, 2003) in CRC was due to changes in VEGF_xxx_b or VEGF_xxx_, the levels of VEGF and VEGF_xxx_b were determined by ELISA and isoform-specific ELISA respectively. Total VEGF was significantly upregulated in CRC (288±48 pg VEGF per mg total protein) compared to normal colon (122±23 pg mg^−1^, *n*=18; *P*<0.01, Wilcoxon Figure 5E). VEGF_xxx_b concentrations, however, were no different in tumour extracts (160±50 pg mg^−1^) than in controls (130±23 pg mg^−1^; *P*=0.96, Figure 5F). Since the total VEGF is the sum of the pro- and antiangiogenic isoforms, the level of VEGF_xxx_ expression was calculated in these samples. The mean VEGF_xxx_ concentration in normal mucosa was not significantly different from zero. In CRC tissue, however, the mean VEGF_xxx_ level was 128±53 pg per mg total protein, indicating that the difference in total VEGF expression was due to an upregulation in VEGF_xxx_ production alone. Thus, there was a shift from predominantly VEGF_xxx_b protein in controls to VEGF_xxx_ in CRC (mean±s.e.m. ratio VEGF_xxx_b/total VEGF: 112±12% in controls, 59±12% in tumours; *P*<0.001 paired *t*-test). Figure 5G shows that the ratio of VEGF_xxx_ to VEGF_xxx_b in the tumours was highly variable with some patients having no excess of VEGF_xxx_, and others up to 57-fold excess of VEGF_xxx_. There was no relationship between Dukes' staging and VEGF_xxx_b concentration or proportion (*P*=0.75; one-way ANOVA, data not shown).

### The switch in expression is part of the malignant transformation program

**Figure 6:**
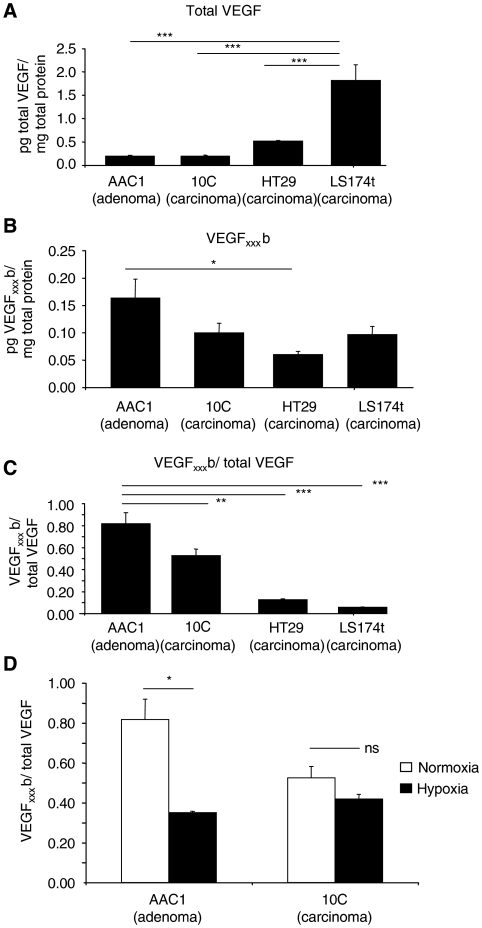
Vascular endothelial growth factor balance shifts from anti- to proangiogenic along the adenoma-carcinoma sequence *in vitro*. HT29, LS174t and 10C well-differentiated adenocarcinoma cell lines and the 10C parent, AAC1 adenoma cell line, were grown to 100% confluence and VEGF expression measured by ELISAs. (**A**) Total VEGF expression increased from adenoma to carcinoma. (**B**) In contrast, VEGF_xxx_b expression decreased along this axis. (**C**) The ratio of VEGF_xxx_b to total VEGF therefore decreased significantly. (**D**) Hypoxia reduced VEGF_xxx_b expression in AAC1 adenoma but not in the 10C carcinoma cell lines. One-way ANOVA with Newman–Keuls *post-hoc* tests, confirmed overall ((**A**): *P*<0.0001; (**B**): *P*<0.0004; (**C**): *P*<0.05, (**D**): *P*<0.001) and individual differences indicated by ^*^P<0.05, ^**^P<0.01, ^***^P<0.001.

As VEGF expression is predominantly proangiogenic in CRC and predominantly antiangiogenic in normal colonic epithelium, cell lines at different stages of the malignant transformation may reflect this. Cells along the adenoma-carcinoma sequence, grown in 100% confluent monolayers, were assayed for VEGF expression as shown in Figure 6. Although the total VEGF expression increased along the adenoma-carcinoma sequence, the degree to which it did so was very variable (Figure 6A) and only the LS174t cells demonstrated a significant such increase. In addition, the VEGF_xxx_b expression was reduced in the carcinoma cells compared to the adenoma cells, but this only reached significance in the HT29 cell line (Figure 6B). Overall, the effect of a shift from anti- to proangiogenic VEGF expression predominance in CRC cell lines was due to a combination of reduced VEGF_xxx_b expression and increased VEGF_xxx_ expression, resulting in a shift from 82±10% VEGF_xxx_b in adenoma cells to between 5.7±0.3 and 53±5.8% in the carcinoma cell lines (*P*<0.0001, one-way ANOVA, Figure 6C). There was thus a predominance of VEGF_xxx_b in this adenoma cell line, and a variable switch towards predominance of VEGF_xxx_ in the colonic carcinoma cell lines.

To test whether hypoxic upregulation of VEGF expression induced a switch in the relative expression of pro- and antiangiogenic VEGF required the use of cell lines that produced predominantly VEGF_xxx_b in normoxia, such as AAC1 and 10C. The exposure of the AAC1 cells to hypoxia resulted in a switch from predominantly antiangiogenic VEGF_xxx_b (82±10% VEGF_xxx_b) to predominantly proangiogenic VEGF_xxx_ (65±1% VEGF_xxx_; *P*<0.01, Figure 6D). This change in the AAC1 cells was due to an upregulation in the proangiogenic isoforms of VEGF, since total VEGF increased (*P*<0.01, Table 1) but VEGF_xxx_b isoforms did not significantly alter. Neither the total VEGF nor the VEGF_xxx_b expression in 10C cells was significantly altered by hypoxia.

### Effect of VEGF_165_b expression on colon cancer growth rates

Prior to injection of the tumour cells into the mice, expression of VEGF isoforms was measured both by western blotting and by ELISA. Vascular endothelial growth factor concentrations of the LS174t human colonic carcinoma cell line transfected to overexpress VEGF_165_, VEGF_165_b, VEGF_165_ and VEGF_165_b (VEGF_165/165_b), or with the empty expression vector (pcDNA3) are given in Table 1.

Proliferation of LS174t cells after transfection with VEGF_165_, VEGF_165_b, both or control vector was measured by flow cytometry. Histogram analysis of propidium iodide-labelled cells showed that there was no significant difference between the cell cycle stage of the groups (control 79±2.3%, VEGF_165_ 80±3%, VEGF_165_b 83±5% and VEGF_165/165_b 77±0.7% in G_0_/G_1_; *P*>0.1). Apoptosis and necrosis were also measured by dual PI/Annexin V staining. There were no significant differences between the four groups (data not shown).

### VEGF_165_b slows LS174t tumour growth *in vivo*

**Figure 7:**
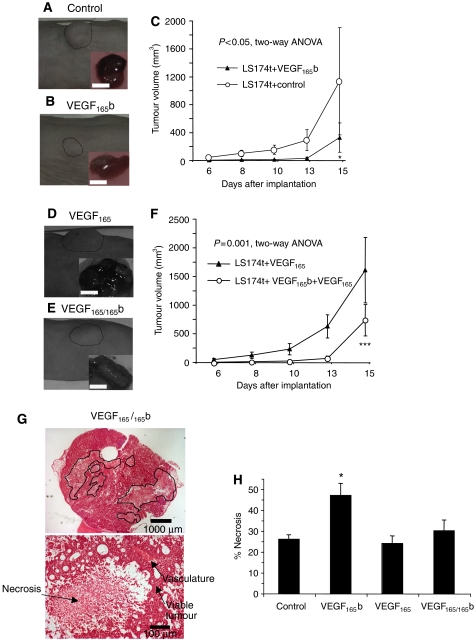
VEGF_165_b inhibits colon cancer growth *in vivo*. (**A**) A total of 2 × 10^6^ LS174t cells transfected with expression vector pcDNA3 injected subcutaneously into nude mice resulted in tumours after 15 days that were large and bloody (excised tumour inset). (**B**) LS174t cells overexpressing VEGF_165_b were smaller and paler. (**C**) Caliper measurements of tumours revealed that VEGF_165_b-expressing tumours grew significantly more slowly than control tumours. (**D**) VEGF_165_-overexpressing LS174t cells injected subcutaneously into nude mice resulted in tumours that were large and very bloody. (**E**) LS174t cells with a balance of VEGF isoforms resulted in smaller, paler tumours than those transfected with VEGF_165_ alone. (**F**) Tumours formed from cells expressing both isoforms grew more slowly than those expressing VEGF_165_ demonstrating that VEGF_165_b inhibits VEGF_165_-mediated tumour growth. (**G**) H&E staining of tumour sections was analysed for necrosis. (**H**) Tumours formed from LS174t cells overexpressing control or VEGF_165_ expression vectors had significantly less necrosis than those expressing VEGF_165_b (^*^P<0.05, ^***^P<0.001).

To determine whether VEGF_165_b expression by the tumour cells inhibited tumour growth *in vivo*, LS174t human colon carcinoma cells stably transfected to express VEGF_165_, VEGF_165_b, both isoforms (VEGF_165_+VEGF_165_b), or a control vector were injected into nude mice. Figure 7A shows that control-transfected cells formed large vascular solid tumours within 15 days. Tumours formed from cells overexpressing VEGF_165_b were smaller and less vascularised (Figure 7B) and had a softer texture. Comparison of tumour volumes made by caliper measurements in live animals showed that VEGF_165_b-expressing cells formed significantly smaller tumours than control cells (*P*<0.01, Figure 7C). Overexpression of VEGF_165_ in LS174t cells resulted in large, vascular tumours (Figure 7D). However, injection of cells expressing both VEGF_165_ and VEGF_165_b resulted in smaller, paler tumours than VEGF_165_ alone (*P*<0.001, Figures 7E and F). VEGF_165_b tumours (0.33±0.22 cm^3^) were significantly smaller than the VEGF_165_-expressing tumours (1.61±0.57 cm^3^; *P*<0.001) at 15 days. Exponential curve fitting to the tumour growth curves was used to calculate the doubling time of the tumour groups. The mean (95% CI) doubling times for pcDNA3, VEGF_165_ overexpression and VEGF_165/165_b overexpression were 2.1 (1.9–2.4), 1.9 (1.9–2.1) and 2.4 (2.2–2.6) days respectively. In comparison, the doubling time for VEGF_165_b overexpression was 3.0 (2.5–3.6) days, which was statistically significantly different (*P*<0.001) from the other groups. After excision, tumour sections were stained with haematoxylin and eosin, and areas of necrosis quantified (Figure 7G). VEGF_165_b-expressing tumours had significantly greater areas of necrosis compared with other groups (Figure 7H; *P*<0.05 ANOVA).

### Bevacizumab can bind VEGF_165_b

**Figure 8:**
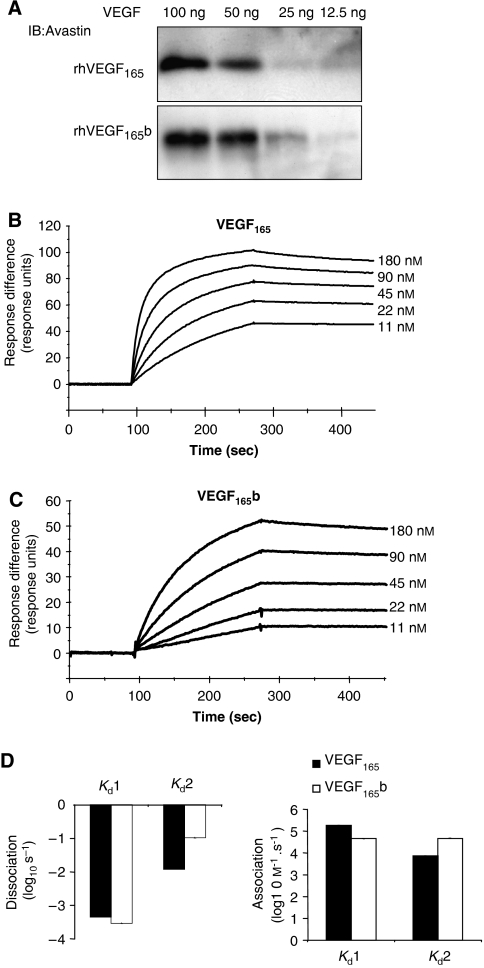
Bevacizumab binds to VEGF_165_b. (**A**) Western blot using bevacizumab as the primary detection antibody. Human recombinant VEGF_165_b or VEGF_165_ was run on a denaturing gel at increasing concentrations. Both isoforms were detected by bevacizumab. Biacore analysis of (**B**) VEGF_165_ and (**C**) VEGF_165_ b binding to bevacizumab. Curves show dose-dependent increase in response units for both VEGF_165_b and VEGF_165_, and dissociation after washout that results in similar binding affinities. (**D**) Mean dissociation and association coefficients for VEGF_165_b and VEGF_165_.

To determine whether bevacizumab bound VEGF_165_b, a western blot of recombinant human VEGF_165_ and VEGF_165_b was carried out by immunodetection with bevacizumab and secondary antibodies to human IgG (Figure 8A). Bevacizumab appeared to detect efficiently recombinant human VEGF_165_b. To determine the efficiency of binding, and its affinity for VEGF_165_b, we carried out Biacore analysis of bevacizumab covalently bound to a surface plasmon resonance sensor. Figure 8B shows the association and disassociation kinetics of VEGF_165_, and Figure 8C those for VEGF_165_b across this chip (Figure 8D). The ratio of these two gives the overall affinity (*K*_D_). The *K*_D_ was similar for VEGF_165_ (2.5 nM) and VEGF_165_b (6.8 nM).

### VEGF_165_b inhibits the effect of bevacizumab

**Figure 9:**
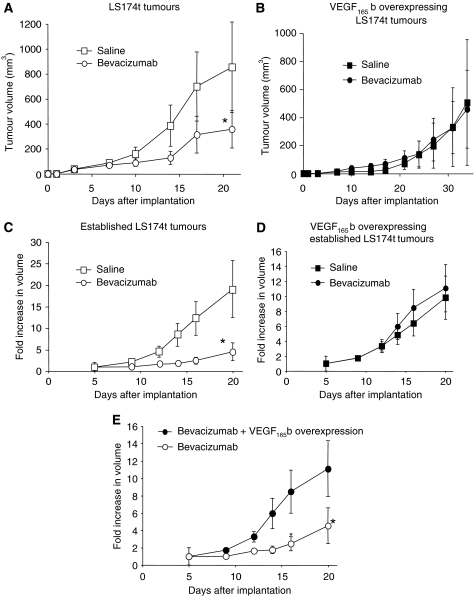
Bevacizumab does not inhibit growth of VEGF_165_b-expressing tumours. LS174t cells overexpressing VEGF_165_b or controls were implanted into nude mice, and treated with twice weekly intraperitoneal injection of either saline or 50 *μ*g of bevacizumab. (**A**) Bevacizumab inhibited growth of LS174t cells compared with saline-injected controls (*P*<0.01). (**B**) In contrast, bevacizumab had no effect on the growth of VEGF_165_b-expressing tumour cells (*P*>0.5). (**C**) LS174t cells overexpressing VEGF_165_b or control LS174t cells were implanted into nude mice, and allowed to form tumours of ∼4 mm diameter prior to treatment by twice weekly intraperitoneal injection of either 50 *μ*g of bevacizumab or saline. Bevacizumab inhibited growth of established tumours compared with saline-injected controls (*P*<0.01). (**D**) In contrast, bevacizumab had no effect on the growth of VEGF_165_b-expressing tumour cells (*P*>0.5). (**E**) VEGF_165_b-expressing tumours grew faster than control tumours when treated with bevacizumab, indicating that VEGF_165_b expression inhibited the effect of bevacizumab (*P*<0.01).

To determine whether the effect of bevacizumab on tumour growth was dependent on VEGF_165_b expression, nude mice were injected with either VEGF_165_b-transfected LS174t colon cancer cells expressing 95% VEGF_165_b (*n*=14), or control LS174t cells expressing 94% VEGF_165_ (*n*=12). Twenty-four hours after tumour cell injection, bi-weekly treatment with 50 *μ*g bevacizumab or saline was started, and tumour sizes measured every 3–4 days. Figure 9A shows that bevacizumab significantly inhibited the growth of VEGF_165_-expressing colon cancer cells (*P*<0.05) within 15 days. Figure 9B shows that even after 35 days of treatment, bevacizumab had no effect on tumour growth in cells expressing predominantly VEGF_165_b. To determine whether the effect of bevacizumab on previously established tumours was modified by VEGF_165_b, cells were injected as before, and tumours allowed to grow to 4 mm in diameter before treatment with bevacizumab as above. Figure 9C shows that bevacizumab inhibited the growth of established tumours compared with saline treatment (*P*<0.05), whereas Figure 9D shows that it did not affect the growth of VEGF_165_b-expressing tumours. Figure 9E shows that VEGF_165_b-expressing tumours grew faster than those not expressing VEGF_165_b when treated with bevacizumab (*P*<0.05).

### VEGF inhibition is toxic to colonic epithelial cells

**Figure 10:**
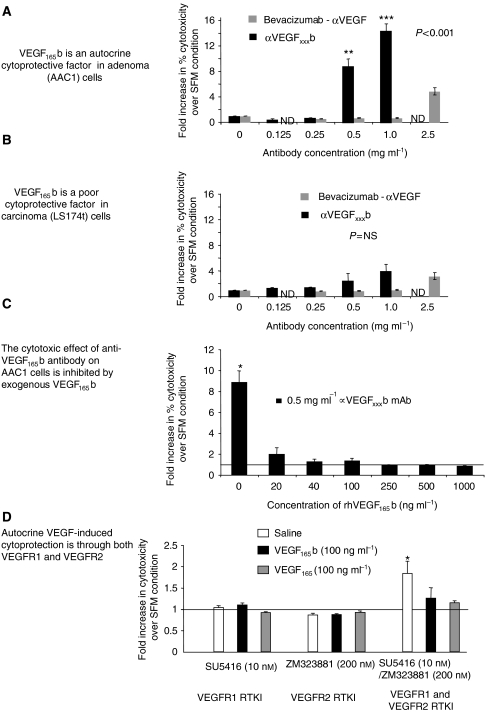
VEGF_xxx_b inhibition is cytotoxic to colonic adenoma cells, but less so to colonic carcinoma cells. Confluent colonic adenoma (AAC1) and carcinoma (LS174t) cells were exposed to serum-free media supplemented with either bevacizumab (anti-VEGF) or anti-VEGF_165_b monoclonal antibodies for 48 h. The media was then harvested prior to cell lysis in fresh media. The two media samples were compared for LDH concentrations, and the ratio used to calculate the relative cytotoxicity. The results were normalised to serum-free media alone and analysed by one-way ANOVA. (**A**) Cytotoxicity was induced in AAC1 cells by antibodies to VEGF_165_b and to bevacizumab. (**B**) LS174t carcinoma cells were less affected by VEGF_165_b blockade than AAC1 cells. (**C**) The cytotoxicity induced by anti-VEGF_165_b antibody was blocked by exogenous rhVEGF_165_b (^***^P<0.0001). (**D**) Autocrine VEGF-induced cytoprotection is through both VEGFR1 and VEGFR2. AAC1 cells were treated with doses of VEGF receptor tyrosine kinase inhibitors previously shown to be specific for VEGFR1 (10 nM SU5416) and VEGFR2 (200 nM ZM323881) and cytotoxicity assayed by LDH ELISA. Inhibiting both VEGF receptors, but neither alone, induced an increase in cytotoxicity. This increase could be abolished with either VEGF_165_b or VEGF_165_. ^*^P<0.05.

VEGF_165_b is strongly expressed in normal colonic tissue and by AAC1 colonic adenoma cells and has been shown to be cytoprotective in renal epithelial cells. Therefore, the sequestration of VEGF_165_b by addition of either a specific anti-VEGF_xxx_b antibody or a more general anti-VEGF antibody could be cytotoxic. To explore this, AAC1 adenoma cells (normally express ∼85% of their VEGF as VEGF_165_b) were treated with either an anti-VEGF_165_b-specific antibody (R&D Systems, cat no. MAB3045) or bevacizumab at increasing doses for 48 h. Cytotoxicity was measured by assaying supernatant for lactate dehydrogenase. Figure 10A shows that VEGF_165_b inhibition was toxic to AAC1 cells in a dose-dependent manner, increasing the cytotoxicity by 14.4±1.1-fold (*P*<0.001). Bevacizumab also significantly increased cytotoxicity 4.9±0.5-fold (*P*<0.001). A nonspecific IgG (1 mg ml^−1^) resulted in a modest 2.2±0.25-fold increase in cytotoxicity over baseline in AAC1 cells (*P*<0.01). Much smaller increases in cytotoxicity were seen when LS174t colonic carcinoma cells were treated with the anti-VEGF_165_b antibody (4.0±0.35-fold) or bevacizumab (3.2±0.6-fold) (Figure 10B, no increase seen with nonspecific IgG). To confirm that the cytotoxicity effects of anti-VEGF antibodies were due to inhibition of VEGF_xxx_b, the effects of supplementing the media with rhVEGF_165_b were measured (Figure 10C). Addition of 40 ng ml^−1^ rhVEGF_165_b protein abolished cytotoxicity induced by the VEGF_165_b antibody (*P*<0.01, one-way ANOVA).

To evaluate whether the VEGF_165_b required for AAC1 cell survival was acting through VEGFR1 or VEGFR2, cells were treated with receptor tyrosine kinase inhibitors to selectively inactivate each of these receptors. Selective inhibition (Glass *et al*, 2006) of either VEGFR1 (10 nM SU5416) or VEGFR2 (200 nM ZM323881) was not toxic to the AAC1 cells (Figure 10D), but combined inhibition of both receptors induced a significant increase in cytotoxicity (1.8±0.29-fold; *P*<0.05, one-way ANOVA), an effect that could be rescued by the addition of 100 ng ml^−1^ of either rhVEGF_165_ or rhVEGF_165_b protein.

## DISCUSSION

Vascular endothelial growth factor has been identified in thousands of studies as being altered in tumours, and able to affect tumour growth. VEGF_165_ was originally identified from tumours and tumour cells, showed angiogenic and propermeability activity and was generated from the sequence now described as exon 8a. In 2002, we described VEGF_165_b, encoded by an alternative sequence in exon 8 (exon 8b), resulting in a protein of identical length but different amino-acid sequence to that encoded by exon 8a. This different C terminus is also found in other VEGF isoforms, resulting in a family of VEGF_xxx_b splice variants (Perrin *et al*, 2005) that is expressed in many normal tissues and mirrors the conventional, angiogenic VEGF_xxx_ isoforms.

### VEGF_165_b is expressed in colonic mucosa

Homodimeric VEGF_165_b is a competitive antagonist of VEGFR2, binding to it with affinity equal to that of VEGF_165_ (Woolard *et al*, 2004). Moreover, VEGF_165_b is not angiogenic, and can act as an antiangiogenic agent in VEGF-mediated angiogenesis, such as in the eye and in the mesentery (Woolard *et al*, 2004). We show here that these antiangiogenic isoforms of VEGF are expressed as mRNA and protein in both normal colonic epithelial cells and colonic carcinomas. Not only is their expression dominant in normal colonic epithelium, but also remains relatively constant in the carcinomas. Many previous studies have identified VEGF upregulation in colon carcinomas either without distinguishing between the two families of isoforms or by methods that only detect the proangiogenic isoforms. Previously described total VEGF levels seen in normal and CRC samples are similar to those described here (40 and 220 pg mg^−1^, respectively (Konno *et al*, 1998)). However, this is a significant underestimate of total VEGF, as the commercially available ELISAs for VEGF have an affinity for VEGF_165_b of only 42% compared with VEGF_165_. Thus, a more accurate estimation of VEGF concentrations in tissues requires a correction for this affinity. Here we show that the measured amount of VEGF_xxx_, but not VEGF_xxx_b, is increased in CRC, supporting the idea that there is a proangiogenic switch involving upregulation of both VEGF overall and, crucially, of only the proangiogenic splice variants.

However, our results show that the VEGF_165_b levels in some tissues (approximately half of the normal samples) exceed those of the total VEGF levels, measured by the pan-VEGF ELISA, even after adjustment for the poor affinity of the pan-ELISA for VEGF_165_b. There are a number of possible reasons for this difference, although none has been clearly proven, mainly relating to the lack of accuracy of the commercial pan-VEGF ELISA when the VEGF_xxx_b isoforms are considered. These include (a) endogenous heterodimerisation, (b) other isoforms and (c) interference by other as yet unknown proteins and fats. Endogenous heterodimerisation may yield intermediate forms such as VEGF_165_b:VEGF_165_ or VEGF_121_b:VEGF_165_b. The affinity of the pan-ELISAs for heterodimers has not yet been shown, but may be intermediate between the two homodimers. If half the VEGF_165_ is dimerised with VEGF_165_b, then the true total VEGF value may be still higher. Other isoforms of VEGF_xxx_b exist (Perrin *et al*, 2005), but the affinity of the ELISAs for these (particularly the commercially available pan-VEGF ELISA) has not been measured. If the affinity of the commercial pan-VEGF ELISA for VEGF_121_b or VEGF_121_ was less than that for VEGF_165_b or VEGF_165_, then this would also result in an underestimate of the total VEGF levels. Finally, the soluble splice variant of VEGFR1 (sFlt-1) has been shown to inhibit detection of VEGF in previous commercially available pan-VEGF ELISAs (Maynard *et al*, 2003). We showed that sFlt-1 does not interfere with the current R&D pan-VEGF ELISA, ruling this out as an explanation for the discrepancy, but other proteins, such as soluble VEGFR2, may affect binding. We show here that VEGF_xxx_b is localised to the same regions of the colonic mucosa as that previously thought to be for proangiogenic VEGF, the lamina propria (Griga *et al*, 2002), goblet cells and glandular cells of the mucosa (Griga *et al*, 1999) and our findings for the pan-VEGF stain are consistent with this. The detection of the same cells by the antibody raised specifically against VEGF_165_b, as by an antibody to all VEGF isoforms, concurs with our ELISA findings that in normal colonic mucosa it is the VEGF_xxx_b isoforms that predominate. This has significant implications for interpretation of all previous studies investigating VEGF expression in the colon, not only for tumour studies (Hurwitz *et al*, 2004), but also for collagenous (Griga *et al*, 2004) and ischaemic colitides (Okuda *et al*, 2005).

### Antiangiogenic VEGF tumour inhibition

The results here show that VEGF overexpression can alter the rate of human xenografted tumour growth *in vivo* and moreover that VEGF_165_b can antagonise the effects of VEGF_165_, thus confirming the role of the C terminus of VEGF in determining its function and the importance of the ratio of VEGF_xxx_b to VEGF_xxx_ in the progression of tumour growth. The ability of AAT to inhibit xenografted tumour growth has been demonstrated previously (Kendall and Thomas, 1993; Kim *et al*, 1993; Kanai *et al*, 1998; Wildiers *et al*, 2003). The effectiveness of AAT has translated into the clinic with bevacizumab in the treatment of renal (Yang *et al*, 2003), breast (Ramaswamy *et al*, 2006) and CRCs (Hurwitz *et al*, 2004). Furthermore, the ability of VEGF_165_b to inhibit VEGF_165_-mediated angiogenesis in a rat mesenteric and rabbit corneal assay (Woolard *et al*, 2004) and mouse retinal angiogenesis assay (Konopatskaya *et al*, 2006) suggests that it may serve as a novel AAT agent. Consistent with this, we show here that the overexpression of VEGF_165_b in human CRC can inhibit tumour growth *in vivo*, further supporting the potential role of relative VEGF_xxx_b downregulation as part of the angiogenic switch in tumour progression. Furthermore, neither VEGF_165_ nor VEGF_165_b overexpression alters the *in vitro* proliferation or apoptosis rates of cells, suggesting that the mechanism of action of VEGF in altering tumour growth rate is not through an autocrine pathway, but likely to be via its known antiangiogenic effects. Furthermore, the antagonistic effects of VEGF_165_b overexpression on tumour growth when co-overexpressed with the potent proangiogenic VEGF_165_ and the increased tumour necrosis observed when VEGF_xxx_b was overexpressed further suggests that VEGF_165_b inhibits tumour growth through antiangiogenesis.

### Bevacizumab binds VEGF_165_b

Bevacizumab binds to all the conventional isoforms of VEGF (Kim *et al*, 1992) via an epitope on the common region of the protein family, adjacent to the receptor-binding site (Muller *et al*, 1997). In CRC, the response to bevacizumab is limited to a small subset of patients, approximately 11–12%, but to date this subset has not been shown to be predictable (Jubb *et al*, 2006). The results here show that the affinity of bevacizumab was similar for VEGF_165_ and VEGF_165_b and concur well with the results by Presta *et al* (1997). Thus the variability in response to bevacizumab could be explained by VEGF_xxx_b expression. The relative expression levels of the VEGF_xxx_ isoforms in human colon carcinoma vary from only 27% of the VEGF_xxx_b isoforms to 60-fold excess, whereas in normal colon, we did not detect excess of VEGF_xxx_ over VEGF_xxx_b. This can explain why in some patients bevacizumab could be a highly effective antiangiogenic agent – those in which the vast majority of the VEGF in their carcinomas is VEGF_xxx_ (the proangiogenic family), whereas in most, those with significant VEGF_xxx_b expression, bevacizumab may be less effective. To date, no biomarkers have been identified that can predict response to bevacizumab (Ince *et al*, 2005; Jubb *et al*, 2006). The differential response in the LS174t tumours to bevacizumab therapy in mice depending upon VEGF_165_b-to-VEGF_165_ ratio shown here is suggestive of its role as a potential biomarker in CRC and possibly other cancers that are responsive to bevacizumab. It remains to be seen whether patients with high levels of VEGF_165_b respond poorly to bevacizumab, but this needs to be tested.

Vascular endothelial growth factor has been shown to be a survival factor for several carcinoma types, including colon. Transfection of RKO colon cancer cells with short interfering RNA against the coding region of VEGF reduced cell proliferation by 67% (Mulkeen *et al*, 2006). However, given that normal colonic epithelial cells produce principally VEGF_xxx_b and the toxicity of blocking VEGF_xxx_b in AAC1 adenoma cells, the autocrine role, may be predominantly due to VEGF_xxx_b in colonic epithelial cells, suggesting for the first time a physiologically active role of VEGF_xxx_b. Furthermore, the general anti-VEGF antibody bevacizumab was also cytotoxic to AAC1 cells, suggesting that bevacizumab could potentially have negative effects on normal colonic epithelium, which expresses high levels of VEGF_xxx_b. A life-threatening complication of bevacizumab therapy is gastrointestinal perforation, the cause of which is unknown (Hurwitz *et al*, 2004), but could be due to its effects on survival of normal colonic epithelial cells, as suggested by the results shown here.

In summary, we show here that in normal colonic epithelium, the antiangiogenic isoforms form the majority of VEGF, and VEGF upregulation in CRC is unique to the proangiogenic isoforms. These results indicate that the role of VEGF in the normal function of the colonic mucosa may depend either on the function of VEGF_xxx_b, which is still unknown, or the effect of the balance between the isoforms. We also show that this switch appears to be an endogenous one to transformation, although environmental cues such as hypoxia can also induce the switch. Furthermore, VEGF_165_b inhibits both colorectal tumour growth (in a VEGF-dependent manner) and the effect of bevacizumab on that tumour growth. We further show that bevacizumab binds VEGF_165_b and that VEGF_165_b is an autocrine survival factor for colonic epithelial cells. These results suggest that anti-VEGF therapy for CRC may be better targeted to patients with significant excess of proangiogenic isoforms over antiangiogenic isoforms, and that therapies that specifically target the proangiogenic isoforms may be more effective.

## Figures and Tables

**Table 1:** VEGF concentrations in transfected cells

	**PcDNA3**	**VEGF_165_b**	**VEGF_165_**	**VEGF_165_b and VEGF_165_**
*LS174t cells fg VEGF per cell per day*				
Total VEGF	4.1±0.4	9.9±1.2^*^	7.0±0.2^*^	35.4±0.2^*^
VEGF_165_b	Undetectable	7.6±0.03^*^	Undetectable	13.3±0.06^*^
VEGF_165_	4.1±0.4	2.3±0.03	7.0±0.2^*^	22.1±0.2^*^

Abbreviation: VEGF, vascular endothelial growth factor.

^*^P<0.05 compared with pcDNA3.
